# Association between the timing of chewable complementary food introduction and functional speech disorders in children: a case-control study

**DOI:** 10.3389/fped.2026.1913696

**Published:** 2026-08-24

**Authors:** Houbin Li, Guilian Huang, Qiang Qi

**Affiliations:** Department of Child Health Care, Hangzhou Fuyang Women and Children Hospital, Hangzhou, Zhejiang, China

**Keywords:** chewable complementary food, child development, complementary feeding, oral motor function, pediatric nutrition

## Abstract

**Objective:**

To investigate the association between the timing of chewable complementary food introduction and functional speech disorders (FSD) in children, and to identify independent risk and protective factors with a primary focus on quantifying the effect of delayed chewable food introduction.

**Methods:**

A case-control study was conducted including 90 children diagnosed with FSD and 90 healthy controls aged 2–8 years. Data were collected on demographic characteristics, electronic screen exposure, parent-child interaction, perinatal risk factors, and complementary feeding practices. Univariate and multivariate logistic regression analyses were performed to assess independent associations.

**Results:**

Delayed introduction of chewable complementary foods (≥12 months) was the strongest independent risk factor for FSD (fully adjusted OR = 35.20, 95% CI: 10.15–122.04, *P* < 0.001). Prolonged screen exposure (>2 h/day) and perinatal high-risk factors were additional risk factors. Female gender and increased parent-child interaction (>2 h/day) were protective factors. Subgroup analyses confirmed the stability of the main association across gender, age, and screen exposure subgroups.

**Conclusions:**

Delayed introduction of chewable complementary foods beyond 12 months is a critical, independent, and modifiable risk factor for childhood FSD. Timely introduction of chewable foods before age 1 year, combined with limited screen time and enhanced parent-child interaction, may serve as a reference to lower FSD risk in early childhood.

## Highlights

- Chewing stimulation supports early orofacial and speech development
- Unreasonable feeding habits link to childhood speech delay
- Screen time and parent interaction affect language development
- Delayed chewable food introduction strongly increases FSD risk
- The association is independent of multiple confounding factors
- Timely textured feeding provides practical clinical prevention advice

## 1 Introduction

Childhood speech disorders are a heterogeneous group of developmental conditions driven by physiological, neurological, and environmental factors (1, 2). Functional speech disorders (FSD) account for 70%–80% of all pediatric speech disorders (3), with a global prevalence of 4%–6% among preschool children (4–6). In China, the detection rate ranges from 5.2% to 8.7% (7). FSD can lead to learning difficulties, social withdrawal, and mental health problems (8, 9).

Oral motor function is essential for normal speech development (10, 11). Chewing stimulates orofacial muscle strength, coordination, and sensory maturation (12, 13). Recent studies suggest a link between inadequate oral motor stimulation and speech disorders (14, 15). A large body of literature has confirmed that sustained masticatory stimulation in infancy promotes coordinated orofacial muscle development, oral sensory maturation and foundational articulatory ability (10, 12, 13). Cross-sectional European and North American cohorts have noted persistent articulation deficits among children fed soft pureed diets long-term (14, 15). Most existing research fails to separate chewable textured meals from general complementary feeding timing, while high-quality epidemiological data focused on Chinese preschoolers remain scarce.

However, large-sample, well-controlled studies on chewable food introduction timing and FSD remain limited. Most prior studies only discuss general complementary feeding timing without distinguishing food texture. Our research specifically targets chewable mastication-required food rather than pureed or liquid supplementary food. We strictly excluded children with hearing impairment, brain lesions and autism to form a homogeneous cohort purely presenting functional speech disorders without organic lesions. Additionally, this study provides localized epidemiological evidence for Chinese preschoolers, while relevant data based on Asian populations is scarce in global literature.

Prior publications carry three core limitations: they do not distinguish chewable texture from pureed complementary food, mixed patients with organic brain/hearing lesions into study cohorts (1, 3), and lack regional pediatric data from Chinese populations (7). Our homogeneous matched case-control design addresses all three research gaps simultaneously. Global authoritative infant feeding guidelines released by WHO recommend progressive introduction of age-appropriate chewable foods between 6 and 12 months to support oral motor maturation (16), which highlights the clinical value of exploring adverse speech outcomes linked to delayed texture transition.

We put forward a testable primary hypothesis: After adjusting for demographic, lifestyle and perinatal confounding factors, delayed introduction of chewable complementary food beyond 12 months is independently positively associated with elevated odds of childhood functional speech disorders. This study aimed to evaluate the independent association between delayed chewable complementary food introduction and FSD, and to identify additional modifiable risk factors.

## 2 Methods

### 2.1 Ethics statement

This study was approved by the institutional ethics committee and conducted in accordance with the Declaration of Helsinki (17). Written informed consent was obtained from the parents or legal guardians of all participating children.

### 2.2 Study population

Participants were recruited between September 2024 and July 2025. All eligible children were consecutively enrolled from pediatric speech outpatient clinics without pre-screening based on feeding history to minimize selection bias. Unified diagnostic and screening checklists were used by two fixed pediatric clinicians during all recruitment procedures. The case group included 90 children diagnosed with FSD using the Goldman-Fristoe Test of Articulation-3 (GFTA-3) (18), which demonstrates reliable articulation assessment performance among Mandarin-speaking preschool children. The control group included 90 healthy children with normal speech development.

Exclusion criteria: structural articulatory abnormalities, hearing impairment, brain injury, autism spectrum disorder, neurological dysarthria.

**Figure 1:**
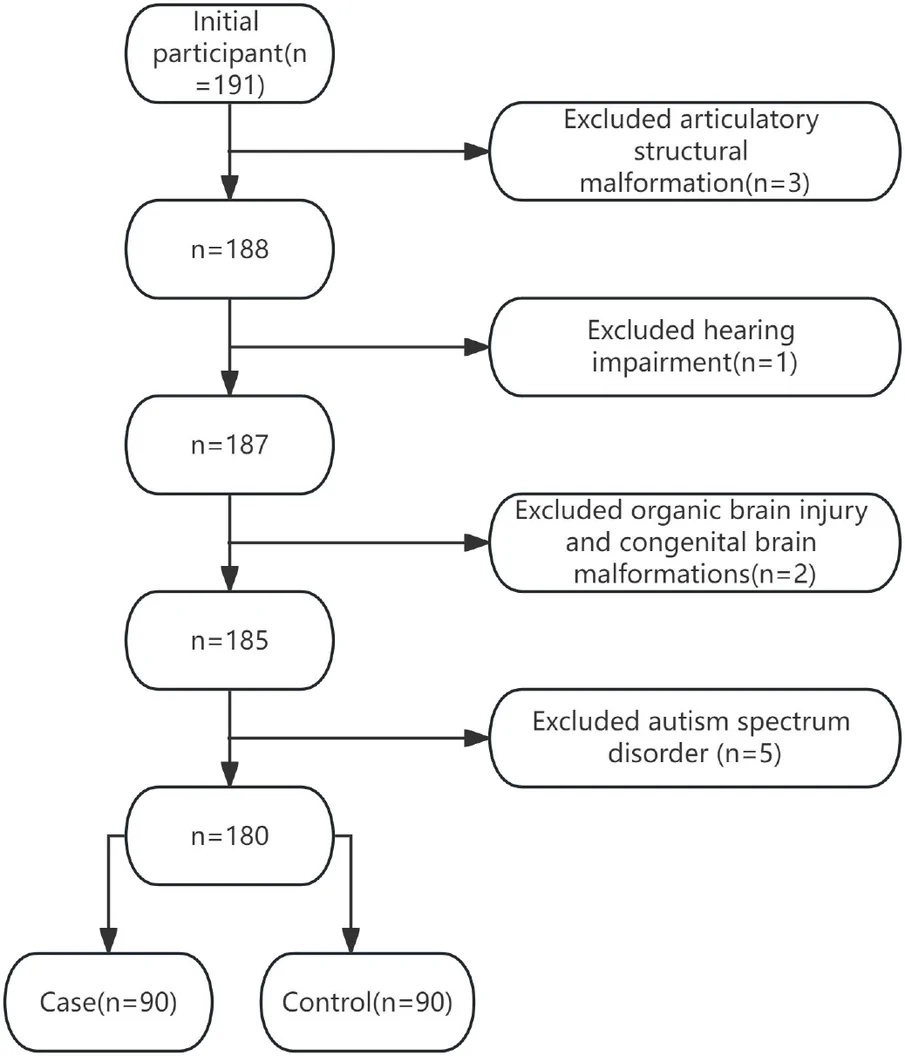
Flowchart of matched case-control participant recruitment and screening. No eligible caregivers refused participation; all questionnaires with incomplete feeding data were excluded before grouping, finally forming separate case (*n* = 90) and control (*n* = 90) cohorts. All exclusion criteria were implemented with unified standards.

Participant recruitment is shown in Figure 1.

### 2.3 Definition of outcomes and indicators

Chewable complementary food introduction timing was categorized as <12 and ≥12 months, based on WHO guidelines (16).

Covariates included gender, age, screen exposure, parent-child interaction, and perinatal high-risk factors (4, 6, 9, 19, 20). All covariates including daily parent-child interaction duration and electronic screen exposure time were collected via standardized one-on-one face-to-face interviews with caregivers, supported by a pre-designed questionnaire. The questionnaire was compiled referring to international validated pediatric feeding and developmental scales, and had undergone reliability and validity testing before subject recruitment. Two trained pediatric clinicians conducted all interviews following fixed questioning scripts to avoid inter-investigator bias. Caregivers completed retrospective reports of children's early daily routines according to unified standardized items, which ensures study reproducibility and allows readers to assess potential measurement bias. The questionnaire passed internal reliability testing before recruitment, yet no historical child health feeding records were available for cross-verification, which inevitably carries risks of parental recall deviation and exposure misclassification.

### 2.4 Statistical analysis

Logistic regression was used to evaluate independent effects. We adjusted all available core confounders including gender, age, electronic screen exposure, parent-child interaction and perinatal high-risk factors in multivariate models. Confounders were retained based on a change-in-effect estimate >10% (21). Subgroup analyses were performed by gender, age, and screen exposure.

Sample size power calculation was completed before participant enrollment. Based on preliminary clinical effect estimation (*α* = 0.05, statistical power = 0.8), a minimum of 86 matched case-control pairs were required; our 90:90 sample fully meets this threshold. Two core logistic regression assumptions were verified before modeling: independent observation of all participants and absence of severe multicollinearity. Confounders were retained if removing them caused >10% change in the primary exposure effect estimate (21). All subgroup stratified models adopted the identical full covariate adjustment scheme as Model II in multivariate analysis.

## 3 Results

### 3.1 Baseline characteristics

Baseline characteristics are presented in Table 1. The case group had significantly higher rates of delayed chewable food introduction, prolonged screen exposure, limited parent-child interaction, male gender, and perinatal risk factors (all *P* < 0.05).

**Table 1:** Baseline characteristics of the study population.

Variables	Total (*n* = 180)	Case (*n* = 90)	Control (*n* = 90)	*P*-value
Gender, *n* (%)				<0.001
Male	117 (65.0)	71 (78.9)	46 (51.1)	
Female	63 (35.0)	19 (21.1)	44 (48.9)	
Age, mean ± SD	4.0 ± 1.4	3.9 ± 1.6	4.1 ± 1.1	0.198
General complementary feeding ≥ 1 year, *n* (%)	8 (4.4)	6 (6.7)	2 (2.2)	0.278
Screen exposure≥2 h/day, *n* (%)	69 (38.3)	52 (57.8)	17 (18.9)	<0.001
Parent-child interaction <2 h/day, *n* (%)	61 (33.9)	40 (44.4)	21 (23.3)	<0.001
Perinatal high risk, *n* (%)	13 (7.2)	10 (11.1)	3 (3.3)	0.026
Delayed chewable food ≥ 1 year, *n* (%)	65 (36.1)	56 (62.2)	9 (10.0)	<0.001

Continuous data expressed as mean ± standard deviation (SD); categorical data as *n* (%). Comparisons by independent *t*-test/chi-square test. FSD, functional speech disorders.

### 3.2 Univariate and multivariate analyses

Univariate analysis (Table 2) showed that delayed chewable food introduction was strongly associated with FSD (OR = 14.82, *P* < 0.001). In the fully adjusted model (Table 3), the association remained highly significant (OR = 35.20, *P* < 0.001).

**Table 2:** Univariate logistic regression analysis of risk factors.

Variable	OR (95% CI)	*P*-value
Female vs. male (gender)	0.28 (0.15–0.54)	<0.001
Age (per year)	0.87 (0.70–1.08)	0.198
General complementary feeding ≥ 1 year	3.14 (0.62–16.01)	0.168
Screen exposure ≥2 h/day	5.88 (3.00–11.52)	<0.001
Parent-child interaction <2 h/day	0.38 (0.20–0.72)	0.003
Perinatal high risk	3.62 (0.96–13.64)	0.057
Chewable food introduction ≥ 1 year	14.82 (6.60–33.32)	<0.001

OR, odds ratio; CI, confidence interval. Reference groups: male, <1 year feeding, <2 h daily exposure/interaction, no perinatal risk.

**Table 3:** Multivariate logistic regression analysis of the timing of chewable complementary food introduction and FSD risk.

Variable	*n* Total	Crude model		Model I		Model II	
		OR (95% CI)	*P* value	OR (95% CI)	*P* value	OR (95% CI)	*P* value
Chewable complementary food introduction							
<1 year	115	1 (Ref)		1 (Ref)		1 (Ref)	
≥1 year	65	14.82 (6.60–33.32)	<0.001	18.73 (7.59–46.21)	<0.001	35.20 (10.15–122.04)	<0.001

OR, odds ratio; CI, confidence interval; Ref, reference group. Model I: adjusted for age and gender. Model II: fully adjusted for all covariates. A two-sided *P* < 0.05 was statistically significant. Socioeconomic covariates including parental education and household income were not collected in this retrospective single-center research, so they could not be incorporated into regression models.

Prolonged screen exposure (OR = 5.88) and perinatal risk factors (OR = 3.62) were additional risk factors. Female gender (OR = 0.28) and increased parent-child interaction (OR = 0.38) were protective.

### 3.3 Subgroup analysis

**Figure 2:**
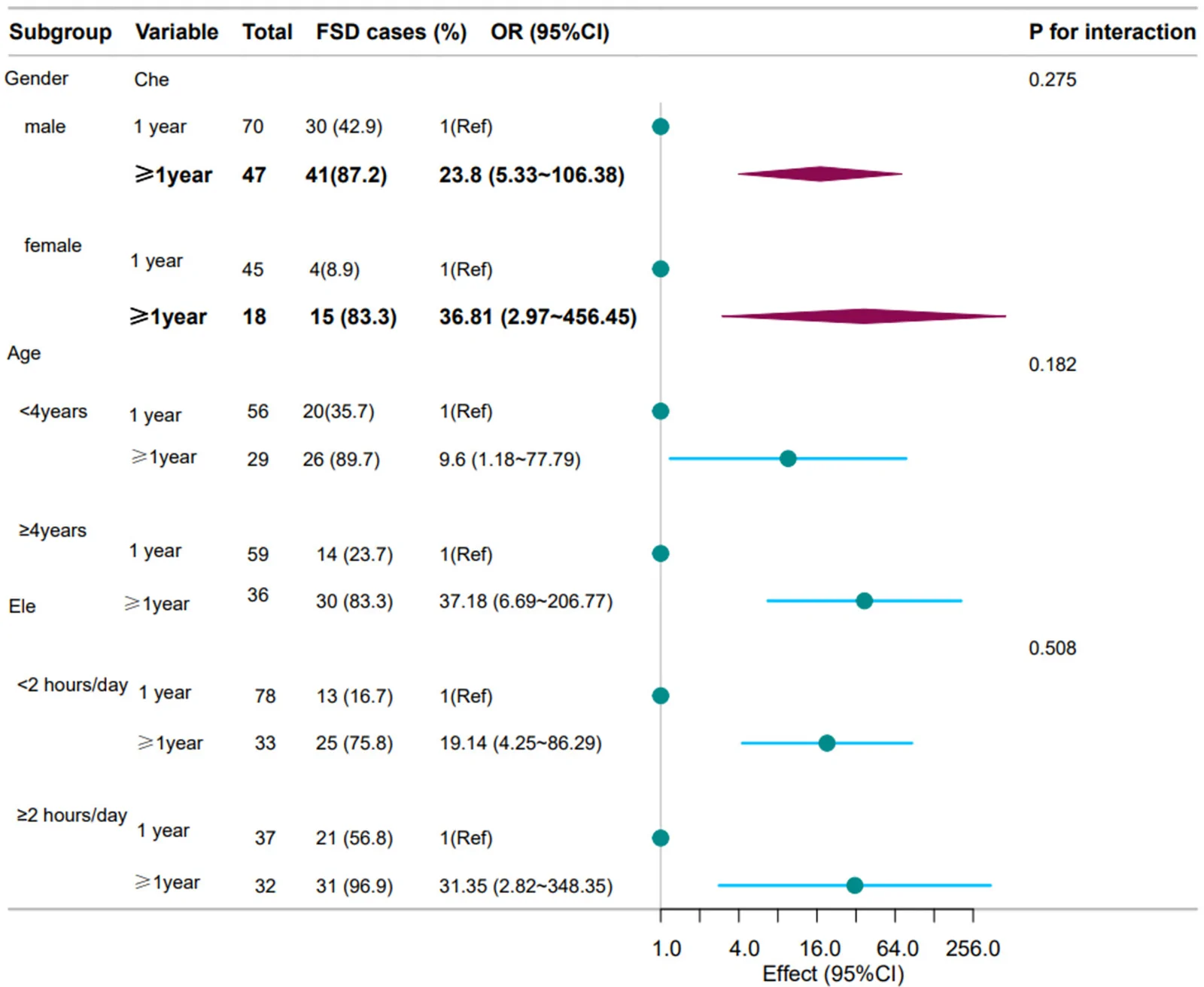
Forest plot of stratified subgroup logistic regression analyses for delayed chewable complementary food introduction (≥12 months) and FSD risk. All subgroup models were fully adjusted for gender, age, daily screen exposure, parent-child interaction time and perinatal high-risk factors; the reference group for chewable food timing was children <12 months old. No statistically significant interaction effects were detected across all strata (all *P* for interaction > 0.05).

Subgroup analyses (Figure 2) confirmed that delayed chewable food introduction was consistently associated with increased FSD risk across all subgroups, with no significant interaction (all *P* for interaction > 0.05).

Sensitivity analyses were further performed to verify the stability of the core association, and detailed results are presented in Supplementary Table 1 (uploaded as a separate supplementary file during submission).

## 4 Discussion

This study's primary finding-that delayed introduction of chewable complementary foods beyond 1 year of age is a strong, independent risk factor for FSD in children (fully adjusted OR = 35.20)-fills a critical knowledge gap in the existing literature by quantifying the magnitude of the association between chewable food introduction timing and FSD risk and establishing its independence after adjusting for a comprehensive set of *a priori* selected confounders (1–3, 12, 14). This finding is consistent with the theoretical basis of our study hypothesis, as chewable food introduction is the primary environmental stimulus for oral motor function maturation in early childhood (10, 12, 13), and impaired oral motor development from delayed chewable food intake directly impairs the coordinated articulatory movements required for clear speech (11, 14, 15). Early introduction of chewable complementary foods (6–11 months) promotes the development of tongue flexibility, mandibular stability, and orbicularis oris muscle strength (15, 18); in contrast, delayed introduction beyond 1 year of age may miss the critical developmental window for oral motor maturation (12, 16), which is correlated with persistent articulatory deficits and elevated FSD risk.

All descriptions of trigeminal neural regulation, brain circuit plasticity and FOXP2 gene expression below are merely theoretical deductions derived from animal and basic human laboratory research (22, 23); our clinical observational study lacks direct neurobiological testing indicators and cannot provide empirical verification of these molecular pathways. The mechanistic link between chewable food introduction and FSD risk involves both orofacial motor maturation and neuroplasticity in language-related brain regions. Repeated masticatory movements stimulate the trigeminal afferent pathway and modulate plasticity in sensorimotor, basal ganglia and frontal-striatal circuits essential for articulatory control and language function (21–23). Accumulated evidence from animal and human studies indicates that oral motor stimulation may influence the expression of the *FOXP2* gene, a key regulator of speech and language development (22, 23), providing a plausible molecular basis for the robust association observed in our research. The notably high adjusted OR (35.20, 95%CI: 10.15–122.04) with a wide confidence interval can be explained by three factors: first, the matched sample size of 90 case-90 control pairs moderately amplifies statistical association magnitude and widens confidence intervals; second, delayed chewable food intake acts as a strong proximal risk factor for oral motor dysfunction within our Chinese pediatric cohort; third, distinct inter-family feeding habits further enlarge between-group differences. Additionally, retrospective self-reported feeding data may generate mild exposure misclassification, and unmeasured socioeconomic confounders may jointly inflate the magnitude of the observed association. Although this association reaches statistical significance, the extremely large OR value cannot be directly extrapolated to general pediatric clinical risk prediction, restricted by single-center retrospective small sample limitations, and requires large prospective cohorts to verify its real clinical reference value. We performed sequential single-covariate exclusion sensitivity analysis to verify model stability. The adjusted OR of delayed chewable food introduction remained persistently elevated (range: 28.63–38.45, all *P* < 0.001) across all models, which confirms the robustness of our primary finding. The wide 95% confidence intervals observed in both overall and subgroup analyses reflect limited matched sample volume and uneven exposure distribution between groups, indicating non-negligible statistical uncertainty of effect estimates. Detailed sensitivity outcomes are summarized in Supplementary Table 1. Our results build upon previous cross-sectional observations linking masticatory dysfunction to speech sound disorders (12, 14, 15), and further confirm that this association remains independent after rigorous adjustment for multiple perinatal and lifestyle confounders.

Our core finding that delayed textured food intake correlates with childhood speech disorders is consistent with published Western cohort conclusions. Obvious discrepancies still exist: Western studies reported much lower odds ratios, attributed to racial feeding culture differences, varied sample sizes and inconsistent covariate adjustment strategies. Most domestic similar researches only adopted simple binary exposure grouping, while our study supplemented stratified subgroup forest plots to verify consistent associations across age, gender and screen exposure strata, further enriching regional clinical evidence.

In the present analysis, prolonged electronic screen exposure and limited parent-child interaction emerged as additional modifiable risk factors, while female gender showed a protective effect, which is consistent with previous population studies (4, 6, 9, 19). Excessive screen exposure may reduce face-to-face communication and language input in early life, and may also reflect insufficient parental attention to age-appropriate feeding arrangements (19, 24). Sustained parent-child interaction enriches linguistic stimulation and is closely correlated with standardized feeding practices (9, 25). The male predominance of FSD observed in our cohort is also in line with existing epidemiological findings (4–6). Notably, these covariates did not attenuate the strong independent association between delayed chewable food introduction and FSD, further highlighting feeding timing as a core intervention target.

Guidance covering age-appropriate chewable food introduction before 1 year of age may be incorporated into routine pediatric developmental follow-up, given its observed correlation with lower odds of FSD in this cohort (16). Pediatric and maternal-child health providers should actively educate caregivers on the importance of early oral motor stimulation, and correct the misconception that prolonged soft-food feeding is safer for young children (26–28). For high-risk subgroups including male infants, children with perinatal complications, and those with excessive screen exposure, targeted oral motor assessment and individualized textured feeding guidance are recommended. Meanwhile, limiting daily screen time and increasing high-quality parent-child interaction can serve as supplementary measures to reduce the overall burden of FSD in early childhood. These low-cost, easily implemented measures are suitable for large-scale popularization in primary child health care.

Several limitations of this study should be acknowledged. First, recall bias is an inherent limitation of this retrospective case-control study, since all early feeding information relied on long-term parental memory. We took multiple measures to reduce memory deviation during data collection: all interviews were completed within 3 months after children's FSD diagnosis to shorten the recall period; clinicians guided caregivers to recall feeding details based on children's key growth milestones; inconsistent statements from fathers and mothers were confirmed through secondary follow-up interviews. Even with these optimized collection methods, subtle memory inaccuracies cannot be fully eliminated, and large prospective cohort studies are required in the future to validate our results (29, 30). Second, we only adjusted all available core covariates collected in this single-center retrospective research, and complete data on parental educational attainment, household monthly income and detailed family language background were not recorded during subject enrollment. These unmeasured speech-related indicators inevitably lead to residual confounding and may bias our association estimates. We will fully collect these socioeconomic and linguistic confounders in our future prospective cohort research to improve analytical rigor (4, 6, 9, 19, 20). Third, all participants were recruited from a single tertiary hospital in Hangzhou, which may limit the generalizability to other regions and cultural backgrounds. Fourth, this study only analyzed the timing of chewable food introduction, without further exploring the influence of food texture type, feeding frequency and feeding quantity. Fifth, the case-control observational design of this study cannot establish definitive causal relationships between delayed chewable food introduction and FSD. Reverse causality cannot be fully ruled out: subtle early speech developmental delays in infants may unconsciously prompt caregivers to adopt long-term soft-food feeding modes, confounding the correlational association we observed. Six, several critical confounding variables were not collected during retrospective enrollment, including infant dietary diversity, daily feeding difficulties, parental educational attainment and household socioeconomic status. These unmeasured indicators may simultaneously affect complementary feeding behaviors and children's speech development, inevitably introducing residual confounding into all regression models. Besides, our study adopted pre-defined binary cut-off points for all exposure indicators during initial data collection without recording continuous graded exposure values. Subdividing variables into three ordered subgroups under the current limited sample size will result in undersized subgroups and insufficient statistical power, so large-sample prospective studies with multi-level exposure recording are needed to further explore dose-response relationships. Additionally, several stratified subgroups contain limited participants, generating wide confidence intervals; hence subgroup findings should only be interpreted as exploratory clues rather than definitive conclusions. Further detailed research is warranted. Despite the above limitations, this study has clear strengths including a well-matched case-control design, comprehensive covariate adjustment, and stable results across subgroup analyses, which support the reliability and clinical reference value of our main conclusion.

## 5 Conclusions

Delayed introduction of chewable complementary foods beyond 12 months was independently correlated with higher odds of childhood functional speech disorders in this case-control cohort. Timely introduction before age 1 year may act as a simple, low-cost reference for early FSD preventive intervention. This work refines the classification of complementary feeding by food texture and supplements Asian clinical evidence, forming its core novelty compared with existing research.

## Data Availability

The raw data supporting the conclusions of this article will be made available by the authors, without undue reservation.
