# Supplementary material for: Association between the timing of chewable complementary food introduction and functional speech disorders in children: a case-control study
Source: Front Pediatr. 2026 Aug 24;14:1913696. doi: 10.3389/fped.2026.1913696 (PMC13547250; doi:10.3389/fped.2026.1913696)
Supplement: Supplementary file 1 [file Table1.doc]

**Supplementary Table 1.** Sensitivity analysis of multivariate logistic regression models to test the stability of the association between delayed chewable complementary food introduction (≥12 months) and functional speech disorders

| Adjusted Covariates in Model | Adjusted OR (95% CI) of delayed chewable food intake | P-value |
| --- | --- | --- |
| Full model (all available covariates) | 35.20 (10.15–122.07) | <0.001 |
| Excluding electronic screen exposure | 32.18 (9.54–108.56) | <0.001 |
| Excluding parent-child interaction time | 34.79 (10.07–120.12) | <0.001 |
| Excluding gender | 28.63 (8.62–95.14) | <0.001 |
| Excluding age | 38.45 (11.23–131.68) | <0.001 |
| Excluding perinatal high-risk factors | 33.91 (9.87–116.52) | <0.001 |

Note: All models adjusted for the remaining confounders after removing the listed single variable one at a time.
